# Association between thyroid autoimmunity and ovarian reserve in women with hypothyroidism

**DOI:** 10.1186/s13044-021-00095-0

**Published:** 2021-03-22

**Authors:** Felipe A. Morales-Martínez, Luis H. Sordia-Hernández, Martha Merino Ruiz, Selene Garcia-Luna, Otto H. Valdés-Martínez, Oscar Vidal-Gutierez

**Affiliations:** 1 Centro Universitario de Medicina Reproductiva, Hospital Universitario “Dr. José Eleuterio González”, Universidad Autónoma de Nuevo León. Monterrey, Edificio Rodrigo Barragán 3er piso Ave., Av. Francisco I. Madero s/n y Gonzalitos Col. Mitras Centro, NL C.P. 64460 Monterrey, Mexico; 2 Departamento de Ginecología y Obstetricia, Hospital Universitario “Dr. José Eleuterio González”, Universidad Autónoma de Nuevo León, NL Monterrey, México

**Keywords:** Antral follicle count, AMH, Hypothyroidism, FSH

## Abstract

**Background:**

The ovarian function and therefore the ovarian reserve may be compromised by the pathogenesis of autoimmune diseases of which, Hashimoto’s thyroiditis (HT) is the most common in women of reproductive age. Furthermore, a prolonged reduction in thyroid hormone concentration results in a broad spectrum of reproductive alteration. Previous reports in the literature have been controversial regarding the impact of hypothyroidism and alterations in the ovarian reserve. Thus, this prospective and comparative study aimed to evaluate the association of hypothyroidism with low ovarian reserve.

**Materials and Methods:**

A subset of 27 patients with primary autoimmune hypothyroidism were compared to healthy women. The ovarian reserve was assessed through the anti-Mullerian hormone (AMH) and the antral follicle count (AFC).

**Results:**

Overall, the two groups did not display significant differences in length of their menstrual cycles neither in the AMH serum levels nor the AFC.

**Conclusions:**

No significant alteration was found in the ovarian reserve of women with HT.

## Background

Autoimmunity is considered to be involved in the pathogenesis of 4–30 % of premature ovarian failure (POF) cases [1]. The alteration of the immune system may cause disruption of the ovarian function or even deletion of follicles causing a reduced ovarian reserve [2–4]. The most common autoimmune disease in women of reproductive age is the thyroid dysfunction with a prevalence of 5–20 % [5–7]. An altered thyroid function affects a wide variety of functions including growth, development and metabolism [8]. Hypothyroidism is originated either by thyroid autoimmune disease (TAID) or by an insufficient iodine intake [9]. It is well known that a prolonged reduction in thyroid hormone concentration results in a broad spectrum of reproductive alteration, including abnormal folliculogenesis, alterations in the ovulation and fertilization rate, and ovarian failure [9–12]. There have been reports of alterations in the menstrual cycle of patients with Hashimoto’s thyroiditis [11] and also, women with thyroid-related diseases have a higher frequency of infertility compared to healthy individuals [9].

The presence of thyroid hormone receptors in the ovary suggests that thyroid hormones are important for ovarian functions [13]. Currently, the physiopathology behind the hypothyroidism on follicular development and the ovarian follicular reserve is not clear and studies using animal models are contradictory. For instance, some reports have claimed that low levels of thyroid hormones produce a significant decrease in basal luteinizing hormone (LH) release resulting in ovarian atrophy [14] or prolonged periods of vaginal diestrus [15, 16]. Other reports showed small changes in LH and follicle-stimulating hormone (FSH) release and the presence of mature follicles and corpus luteum [17]. In humans, some studies support the relationship between hypothyroidism and a diminished ovarian reserve (DOR) [18, 19]. The DOR is defined by a reduced response to ovarian stimulation in women of reproductive age with regular menstrual cycles when compared to women of equivalent age.

The ovarian reserve (OR) can be accurately assessed by the measurement of the anti-Müllerian hormone (AMH), which is secreted by the granulosa cells of ovarian follicles [20, 21] and which levels are stable throughout the cycle. There is preliminary evidence that thyroid disorders are associated with low OR [22] and also of a possible association between TAID and a DOR [23]. Additionally, the prevalence of DOR in patients with low levels of thyroid hormones is not fully established. The aim of this prospective and comparative study was to evaluate if women with TAID but being euthyroid on replacement therapy have lower ovarian reserve.

## Materials and Methods

### Subjects and samples

 This study was approved by the Bioethics Committee of the Hospital Universitario “Dr. José Eleuterio González” (#GI07-020) and complied with the Declaration of Helsinki principles. Patients were provided with written and verbal information about the study before consenting to participate in the study. We performed a comparative and prospective study that included women of reproductive age (20–35 years old) with primary autoimmune hypothyroidism.

These patients displayed positive anti-TPO antibodies, high levels of thyroid-stimulating hormone (TSH) and low levels of free thyroxine (FT4). The reference values were 0.27–4.2 mIU/l and 9.3–17.0 ng/l (12–23.3 pmol/l) for TSH and FT4, respectively. At the time of the study, the patients were euthyroid due to the administration of thyroid hormones. The average age of patients when hypothyroidism was diagnosed was 25.6 years. The length of replacement therapy with L-thyroxine was 27.04 ± 8.04 months. Positive thyroid antibodies were found at least 1 year before the initiation of this study. A group of patients with normal levels of thyroid hormones and negative for anti-TPO antibodies was included as a control. Patients with history of endometriosis, Poly Cystic Ovarian Syndrome (PCOS), hysterectomy, oophorectomy or any other ovarian surgery, or with the usage of hormonal replacement for menopausal symptoms at the initiation of the study or during follow-up; or taking hormonal contraception for ≥ 3 months before the study onset was not included in the study. The body mass index (BMI) was calculated using the formula BMI = Weight (Kg)/Height (m^2^). According to the BMI, individuals with values between 18.5 and 24.5 were classified as normal weight whilst a BMI of 25 and 29 were considered overweight and above 30 as obese.

### Transvaginal ultrasonography

Transvaginal ultrasonography with the Voluson Expert 730 with a 7.5 vaginal transducer (GE Healthcare) was used for the determination of the AFC and the ovarian volume on the third day of the menstrual cycle. Briefly, once the individual was with an empty bladder and in a lithotomy position, the transducer was advanced about 6 to 8 cm into the vagina angling laterally until the ovary was visualized. The length and Antero-Posterior (AP) measurements were obtained in the longitudinal plane, while in the transverse plane, the antral follicles measuring 2–10 mm in diameter in both ovaries were counted for the AFC.

### Measurement of anti‐mullerian hormone

 After overnight fast, blood samples were taken from the participants. Briefly, blood was drawn into plain serum tubes, centrifugation at 3500 rpm for 5 minutes was performed within 1 h of blood collection and the serum was separated and stored at -20°C until analysis. Serum anti-Mullerian hormone (AMH) levels were assessed by the Mullerian Inhibiting Substance/Anti-Mullerian (MIS/AMH) enzyme linked immunosorbent assay (ELISA) test (Diagnostic Systems, Workingham, UK) following the manufacturer recommendations. The assay demonstrated stable intra- and inter-assay coefficients of variation of 5 % and a functional sensitivity of 0.35 ng/ml.

### Calculations and statistical analysis

Data are expressed as mean ± standard deviation (SD). Statistical comparisons were performed by Student t-test. Statistical analysis was performed with GraphPad Prism version 4.0 for Windows (GraphPad Software, San Diego, CA, USA). A probability (*p*) value *p* < 0.05 was considered statistically significant.

## Results

A total of 52 patients were enrolled in this study, of which, 27 were diagnosed with TAID and 25 displayed normal levels of thyroid hormones and therefore used as a control group. The general features of the patients are shown in Table 1. The age of both groups was not significantly different. In contrast, the TAID group showed higher values of BMI (*p* = 0.02) compared to the control group.

**Table 1:** Description of the study participants

Parameter	TAID (*n* = 27)	Control (*n* = 25)	*p*-value
Age (years)	27.70 ± 4.71	26.2 ± 3.02	0.18
BMI (Kg/m^2^)	27.20 ± 6.69	23.84 ± 3.23	0.02
Length of menstruation (days)	4.59 ± 1.15	3.92 ± 0.70	0.01
Minimum cycle length (days)	29.00 ± 5.13	29.00 ± 1.05	> 0.99
Maximum cycle length (days)	41.00 ± 9.73	31.44 ± 1.95	0.11

The length of the menstrual cycle was longer in patients with hypothyroidism (4.59 ± 1.15) than in the control group (3.92 ± 0.702). However, no differences were observed in the minimum and maximum cycle length between both groups (Table 1).

We used two approaches to estimate the OR, the AMH and the AFC. The basal FSH levels were not different between the TAID and control group with 4.78 ± 2.59 and 4.37 ± 1.96, respectively (Table 2). Similarly, there was no difference in the AMH levels between both groups. Accordingly, no significant differences were found for the AFC and the ovary volume when the groups were compared.

**Table 2:** Clinical parameters by study group

Parameter	TAID (*n* = 27)	Control (*n* = 25)	*p*-value
FSH (IU/L)	4.78 ± 2.59	4.37 ± 1.96	0.52
AFC	7.09 ± 2.18	7.98 ± 1.16	0.07
Ovary volumen	8.16 ± 3.39	8.99 ± 0.72	0.23
AMH (ng/ml)	2.42 ± 1.86	2.77 ± 2.50	0.57

## Discussion

The importance of thyroid hormones for ovarian function has been extensively revised recently and the authors concluded that abnormal levels of thyroid hormones, especially during puberty and fertile age, might result in ovarian dysfunction throughout the entire life [19]. In this prospective study, we observed that there is no significant alteration in the ovarian reserve of women with TAID. Patients with a diminished ovarian reserve presented higher levels of TSH suggesting that thyroid disorders are associated with the ovarian reserve [24]. Other studies have shown the presence of anti-thyroid antibodies in ovarian follicular fluid from women with TAID which were positive correlated with their levels on serum [25]. However, evidence on the correlation between TAID with a low ovarian reserve is controversial [23, 26–28]. Osuka and coworkers [29] reported that thyroid autoantibodies are not likely to influence ovarian reserve in euthyroid women whose TSH levels are in the normal range. In a similar study, Ke et al. [30] analyzed the association between TAID and DOR and found that the presence of antibodies had no impact on ovarian reserve in euthyroid women. They also observed that these autoantibodies do not impact of the ART outcome, similar to what others observed in a subset of patients undergoing ART [9].

The analysis of our results, however, requires an important consideration that need to be taken into account. It was reported that after LT4 supplementation, the AMH level in patients with thyroglobulin antibody-positive and thyroid peroxidase antibody-negative significantly increased [31]. This possibility may introduce a bias in our observations, However, the limited number of patients that were included in Kuroda et al. [31] required that this possibility is explored in larger subsets of patients. Another important consideration is related to the association that has been found between ovarian reserve markers and body mass index (BMI) [32]. In this regard, we have not found a significant association between both parameters in the control group or in the patients (Pearson correlation coefficient of 0.22 and − 0.14, respectively).

The pathophysiological mechanism of the association of the TAID with the ovarian reserve is not completely elucidated. It is proposed that TPO antibodies pass through the blood follicle barrier during follicular development which damages the growing follicles and oocytes. Hypothyroidism patients can have alterations in the menstrual cycle where the most common alteration is oligomenorrhea [9]. In our study, the duration of the menstrual cycles was different among groups despite that all the patients were euthyroid through medication at the time of evaluation.

A study by Chen et al. demonstrated that low ovarian reserve with lower serum concentration of AMH was associated with more frequent positive TPO Ab rather than thyroid function or Tg Ab positivity [27]. The thyroglobulin antibodies were not assessed in this study and the TAID was determined based on the serum TPO Ab measurement.

Several approaches are used to evaluate the ovarian reserve, including the FSH, E2, inhibin B, AMH, the AFC and the ovarian volume (by ultrasound), the clomiphene test, and the exogenous FSH test. Even though we did not assess the inhibin B, the AMH has been indicated to be a reliable biomarker for the ovarian reserve since its levels are constant through the cycle [33].

Finally, although our results did not show significant differences between TAID patients and the control group, it is important to consider the existence of preantral follicles that are not visualized by ultrasound that can produce AMH. Thus, it would be important to assess the ovarian reserve (AMH and AFC) in individuals with primary hypothyroidism before the initiation of the treatment. Our findings also indicate a lack of fertility affectation at the time of the OR assessment. However, the monitoring of the OR through the disease progression is important to provide fertility advice to these patients in a timely manner.

## Data Availability

Available.
